# Exploring the impact of air pollution on COVID-19 admitted cases: Evidence from vector error correction model (VECM) approach in explaining the relationship between air pollutants towards COVID-19 cases in Kuwait

**DOI:** 10.1007/s42081-022-00165-z

**Published:** 2022-06-28

**Authors:** Ahmad R. Alsaber, Parul Setiya, Ahmad T. Al-Sultan, Jiazhu Pan

**Affiliations:** 1 Department of Management, American University of Kuwait, Salmiya, Kuwait; 2 Department of Agrometeorology, College of Agriculture, G.B.Pant University of Agriculture and Technology, Pantnagar, Uttarakhand India; 3 Department of Community Medicine and Behavioural Sciences, College of Medicine, Kuwait University, Kuwait City, Kuwait; 4 Department of Mathematics and Statistics, University of Strathclyde, Glasgow, G1 1XH UK

**Keywords:** Air pollution, COVID-19, Cointegration, Long-run relationship, Kuwait

## Abstract

In urban areas, air pollution is one of the most serious global environmental issues. Using time-series approaches, this study looked into the validity of the relationship between air pollution and COVID-19 hospitalization. This time series research was carried out in the state of Kuwait; stationarity test, cointegration test, Granger causality and stability test, and test on multivariate time-series using the Vector Error Correction Model (VECM) technique. The findings reveal that the concentration rate of air pollutants ($\hbox {O}_3$, $\hbox {SO}_2$, $\hbox {NO}_2$, $\hbox {CO}$, and $\hbox {PM}_{10}$) has an effect on COVID-19 admitted cases via Granger-cause. The Granger causation test shows that the concentration rate of air pollutants ($\hbox {O}_3$, $\hbox {PM}_{10}$, $\hbox {NO}_2$, temperature and wind speed) influences and predicts the COVID-19 admitted cases. The findings suggest that sulfur dioxide ($\hbox {SO}_2$), $\hbox {NO}_2$, temperature, and wind speed induce an increase in COVID-19 admitted cases in the short term according to VECM analysis. The evidence of a positive long-run association between COVID-19 admitted cases and environmental air pollution might be shown in the cointegration test and the VECM. There is an affirmation that the usage of air pollutants ($\hbox {O}_3$, $\hbox {SO}_2$, $\hbox {NO}_2$, $\hbox {CO}$, and $\hbox {PM}_{10}$) has a significant impact on COVID-19-admitted cases’ prediction and its explained about 24% of increasing COVID-19 admitted cases in Kuwait.

## Introduction

Healthcare systems must have sufficient resources to meet demand from COVID-19 cases during the epidemic. One of the most essential planning measures is to examine the association between the daily cases of COVID-19 patients to the concentrations of five major air pollutants: Ozone ($\hbox {O}_3$), sulfur dioxide ($\hbox {SO}_2$), carbon monoxide ($\hbox {CO}$), nitrogen dioxide ($\hbox {NO}_2$), and particulate matter ($\hbox {PM}_{10}$). Only a few articles use leading indicators within multivariate time-series models (Nguyen et al., 2021). We used a multivariate framework called the Vector Error Correction Model to create 30-day-ahead forecasts using a leading indicator, the local COVID-19 infection incidence, as well as the rising or decreasing level of daily concentrations of air pollutants ($\hbox {O}_3$, $\hbox {SO}_2$, $\hbox {NO}_2$, $\hbox {CO}$, and $\hbox {PM}_{10}$). This model is also used to generate 60-day scenario estimates based on various pandemic trajectories. The two-time-series show a steady long-run relationship, according to our findings. In comparison to a more traditional model based solely on medical data, the model exhibits a strong fit for the data and good forecasting performance. Our study proposes a novel model for precise short-term forecasts and practical scenario-based long-term forecasts of COVID-19 daily cases in Kuwait utilizing daily air pollution concentrations ($\hbox {O}_3$, $\hbox {SO}_2$, $\hbox {NO}_2$, $\hbox {CO}$, and $\hbox {PM}_{10}$) to aid healthcare decision-making.

The need for hospital administrators to have timely and precise air pollution projections to plan for surges in hospital demand due to the epidemic spurred our effort. When hospitals surpass their historical capacity, adequate preparation can help minimize or mitigate demands on hospital resources COVID-19. As a result, a model that predicts the number of COVID-19-positive patients who will be admitted to a hospital or health system in the short and long term is critical. This COVID-19 hospital census is vital for making decisions that involve a lot of forethought, such as hiring more people, building physical beds and rooms, and purchasing critical equipment (for instance, personal protective equipment and ventilators).

Using univariate time-series models such as Seasonal Autoregressive Integrated Moving Average (SARIMA), Autoregressive Integrated Moving Average (ARIMA), and exponential smoothing, past research has shown the utility of forecasting hospital demands (e.g., hospital admissions, intensive care unit census, and overall hospital census) (Capan et al., 2016; Earnest et al., 2005; Jones et al., 2008; Konarasinghe, 2020; Nguyen et al., 2021; Roy et al., 2021; Tyagi et al., 2020; Yonar et al., 2020).

In this paper, we seek to acquire further evidence to establish a link between air pollution concentrations of $\hbox {O}_3$, $\hbox {SO}_2$, $\hbox {NO}_2$, $\hbox {CO}$, and $\hbox {PM}_{10}$ with daily COVID-19 admitted cases in Kuwait. Our study’s essential contribution and innovation are as follows:

- To our knowledge, the majority of the existing literature focuses on examining the relationship between COVID-19 admitted cases and other climatology factors like average humidity (Fareed et al., 2020) or examine the correlation between the average daily temperature and the rate of coronavirus epidemic growth in the affected regions (Pirouz et al., 2020). However, there is a paucity of literature that examines the association between daily COVID-19 admitted cases and air pollution.
- Most notably, this study seeks to explore the dynamic causality between air pollutants ($\hbox {O}_3$, $\hbox {SO}_2$, $\hbox {NO}_2$, $\hbox {CO}$, and $\hbox {PM}_{10}$) concentrations rate and the daily COVID-19 admitted cases using the panel Granger causality test based on the vector error correction model (VECM).
- The VECM was chosen for this study for the following reasons: The method can allow endogenous variables; the VECM methodology can provide alternative analysis channels to analyze causality that is disregarded by the traditional Granger causality test due to the error correction term (ECM) (Azlina et al., 2014). Meanwhile, the VECM is capable of distinguishing between short-run and long-run causality (Azlina et al., 2014).

## Literature review

### Relationship between air pollution and human health

Numerous studies have indicated the major air pollutants causing adverse health effects in Saudi Arabia including $\hbox {O}_3$, $\hbox {SO}_2$, $\hbox {NO}_2$, $\hbox {CO}$ and $\hbox {PM}_{10}$ (Al Mulla et al., 2015; Argyropoulos et al., 2016).

It has been discovered that incomplete burning of Arabian incense produces emissions of $\hbox {CO}$, $\hbox {PM}_{10}$, $\hbox {PM}_{2.5}$, black carbon, and polycyclic aromatic hydrocarbons (PAHs), all of which have negative health effects on the population who are exposed to these emissions (Du et al., 2018). Ischemic heart disease (IHD), chronic obstructive pulmonary disease (COPD), and lung cancer have all been linked to these air pollutants (Amoatey et al., 2018).

### Impact of air pollution as risk factor to COVID-19 patients

During COVID-19, air pollution was identified as a risk factor in several Italian research. Among the areas of Northern Italy, a correlation with higher levels of pollutants such as PMs has a considerable impact on human health (Domingo et al., 2020; Martelletti & Martelletti, 2020). It has also been discovered that people who live in areas with high levels of air pollution are more likely to acquire chronic respiratory illnesses and are more susceptible to any infectious agent (Distante et al., 2020).

In China, air pollution has been proven to be positively associated with SARS mortality (Cui et al., 2003). Although COVID-19 risk factors are still being investigated, it is probable that environmental variables such as air pollution could substantially impact the epidemic’s spread among the population. In the case of SARS-CoV-2, many studies have found a significant relationship between air pollution and the rate at which the virus spreads. Six air pollutants ($\hbox {PM}_{2.5}$, $\hbox {PM}_{10}$, $\hbox {SO}_2$, $\hbox {CO}$, $\hbox {NO}_2$, and $\hbox {O}_3$) were significantly linked to confirmed cases in 120 Chinese cities from January 23 to February 29, 2020, according to the Zhu et al. (2020). The most badly afflicted region in Europe is the same as the one with the highest concentrations of $\hbox {PM}_{10}$ and $\hbox {PM}_{2.5}$, according to Martelletti and Martelletti (2020). In addition, the majority of fatality cases were in areas with the highest $\text {NO}_2$ concentrations (Ogen, 2020). According to Bashir et al. (2020) and Sharma et al. (2020), the associations were also confirmed in California, the United States, and India.

#### The relationship between atmospheric variables and COVID-19 cases

Finally, for other coronavirus epidemics, it is well documented in the literature how climatic circumstances can influence transmission, either promoting or reducing it. Atmospheric variables such as ambient temperature and humidity, as well as sun irradiation, have various impacts on coronavirus survival, for example, Casanova et al. (2010) and Lauc et al. (2020). This indicates that the coronavirus spread is facilitated in dry and cold weather. Nonetheless, it is still unknown if and how the SARS CoV-2 virus spreads or is impacted by meteorological factors like other seasonal viruses. Several recent studies looked at the role of meteorological variables in COVID-19 transmission all over the world. As shown in Pani et al. (2020), studies from China (Liu et al., 2020; Ma et al., 2020; Shi et al., 2020; Xie & Zhu, 2020), Iran (Ma et al., 2020), Spain (Briz-Redón & Serrano-Aroca, 2020), USA (Bashir et al., 2020; Gupta et al., 2020), Indonesia (Tosepu et al., 2020), Norway (Menebo, 2020), and also over the global (Sobral et al., 2020; Wu et al., 2020) are controversial and The World Health Organization (WHO) has stated that more research should be focus on how to quantify how the weather affects the virus’s spread.

### Time-series analysis to predict COVID-19 cases

It is clear from previous research that time-series models such as exponential smoothing, ARIMA, and SARIMA performed well and provided adequate results for COVID-19 prediction. Many scholars have researched COVID-19 virus infection predictions. All previous research has established that the ARIMA model is the most effective for forecasting (Benvenuto et al., 2020; Jain et al., 2021; Murugesan et al., 2020; Mustafa & Fareed, 2020; Sahai et al., 2020; Sulasikin et al., 2020). Sulasikin et al. (2020) used three approaches to predict the COVID-19 instances (Holt’s method, Holt–Winters method, and ARIMA). Among the other models, the ARIMA model was deemed the best by the author. Furthermore, Nguyen et al. (2021) demonstrated that the COVID-19 infection incidence could be effectively incorporated locally into a VECM with the COVID-19 hospital data to improve the existing forecast models and produce precise short-term forecasts and practical situation-based long-term trajectories.

## Theoretical notions and the model

The vector autoregressive model (VAR) for analysis was used to evaluate the hypothesis of the influence of industrial pollution on public health. VAR was chosen, because it does not need the assumption of exogeneity of variables a priori and allows each variable to self-interact and interact with other variables without imposing a theoretical structure on the estimates. A Vector Error Correction Model (VECM) is used to approximate the impulse response functions if all variables in our VAR cointegrate with order *I*(1) and if there are cointegration associations between them. The following multivariate model was studied in the study utilizing the VECM to test the long-run associations.

The cointegration rank in VECM indicates the number of cointegrating vectors. A rank of two, for example, suggests that two linearly independent combinations of the non-stationarity variable are stationary. Any short-term variations between the independent variables and the dependent variable will create a stable long-run relationship between the variables if the error correction model (ECM) coefficient is negative and significant.

### Data and variables

The data utilized for the study span the months of March 10, 2020 and December 31, 2020. Kuwait Environment Public Authority provided statistics on air pollutants ($\hbox {O}_3$, $\hbox {SO}_2$, $\hbox {NO}_2$, $\hbox {CO}$, and $\hbox {PM}_{10}$) (K-EPA). Kuwait’s Ministry of Health provided the daily COVID-19 cases (MOH). (https://corona.e.gov.kw/en) presented summary of the daily COVID-19 cases in Kuwait. All of the variables were converted to their natural logarithms before using the model.

### Air Quality Index (AQI)

The Air Quality Index (AQI) is a numerical indicator of a region’s air quality. The AQI scale has the range 0 to 500, with a higher AQI value indicating poor air quality and a lower AQI ($<100$) signifying good air quality in a given area. AQI values were calculated using 24-h average $\hbox {PM}_{10}$ and $\hbox {PM}_{2.5}$, 8-h average $\hbox {CO}$ and $\hbox {O}_3$, and 1-h average $\hbox {NO}_2$ and $\hbox {SO}_2$ levels in the current study. The maximum AQI observed for a city was used as the overall AQI.

### Stationarity test

The ability of a series’ stationarity to impact its behaviour is a significant phenomenon. If the *x* and *y* series are non-stationary random processes (integrated), modelling the *x* and *y* relationship as a simple OLS relationship, as in Eq. (1), will result in a misleading regression

1 $$\begin{aligned} Y_{t}=\alpha +\beta X_{t}+\varepsilon _{t}. \end{aligned}$$

The statistical features of a series over time, such as its mean and variance, are known as time-series stationarity. The series is considered to be a stationary process (that is, not a random walk/has no unit root) if both are constant across time; otherwise, the series is defined as a non-stationary process (that is, a random walk/has unit root) [see Eq. (2)]

2 $$\begin{aligned} \begin{array}{ll} x \text { level } &{}\quad x_{t} \\ x 1{\text {st-differenced value}} &{}\quad \nabla x_{t} = x_{t}-x_{t-1} \\ x 2{\text {nd-differenced value}} &{}\quad \nabla ^{2} x_{t}=\left( \nabla x_{t}-\nabla x_{t-1}\right) =x_{t}-2 x_{t-1}+x_{t-2}. \end{array} \end{aligned}$$

If a series is stationary without any differencing, it is designated as *I*(0), or integrated of order 0. On the other hand, a series that has stationary first differences is designated *I*(1), or integrated of order one (1). Augmented Dickey–Fuller test suggested by Dickey and Fuller (1979), and the Phillips–Perron test recommended by Phillips and Perron (1988) have been used to test the stationarity of the variables.

### Unit-root test

Spurious regression can be reduced by conducting a unit-root test for each variable before analysis, because data are used as an all-time-series. Phillips–Perron (PP), Dickey–Fuller (ADF), and Kwiatkowski–Phillips–Schmidt–Shin (KPSS) tests were used, and the results are available in Tables 4, 5, 6, and 7. Non-stationary variables in an estimated model can lead to spurious results that cannot be used for inferences. The PP test does not assume homoscedasticity in the error term, but this assumption is required for the ADF test.

#### Dickey–Fuller tests (DF test and ADF test)

Dickey–Fuller test (Dickey & Fuller, 1979) is one of the best known and most widely used unit-root tests. It is based on the model of the first-order autoregressive process (Box et al., 1970)

3 $$\begin{aligned} y_{t}=\phi _{1} y_{t-1}+\varepsilon _{t}, \quad t=1, \ldots , T, \end{aligned}$$

where $\phi _1$ is the autoregression parameter, and $\varepsilon _{t}$ is the non-systematic component of the model that meets the characteristics of the white noise process. The null hypothesis is ${\mathrm {H}}_{0}: \phi _{1}=1$, i.e., the process contains a unit root, and therefore, it is non-stationary, and is denoted as *I*(1), alternative hypothesis is ${\mathrm {H}}_{1}:\left| \phi _{1}\right| <1$, i.e., the process does not contain a unit root and is stationary, *I*(0). To calculate the test statistic for DF test, we use an equation that we get if $y_{t1}$ is subtracted from both sides of Eq. (3)

4 $$\begin{aligned} \varDelta y_{t}=\beta y_{t-1}+\varepsilon _{t}, \end{aligned}$$

where $\beta =\phi _{1}-1$. The test statistic is defined as

5 $$\begin{aligned} t_{{\mathrm{DF}}}=\frac{{\hat{\phi }}_{1}-1}{s_{{\hat{\phi }}_{1}}}, \end{aligned}$$

where ${\hat{\phi }}_{1}$ is a least square estimate of $\phi _{1}$ and $s_{\hat{\phi _{t}}}$ is its standard error estimate. Under the null hypothesis, this test statistic follows the Dickey–Fuller distribution, and critical values for this distribution were obtained by a simulation and have been tabulated in Dickey (1976) and Fuller (1976).

Model (3) can be expanded by a constant or a linear trend

6 $$\begin{aligned}&y_{t}=\beta _{0}+\phi _{1} y_{t-1}+\varepsilon _{t}\nonumber \\&y_{t}=\beta _{0}+\beta _{1} t+\phi _{1} y_{t-1}+\varepsilon _{t}. \end{aligned}$$

In the case when a non-systematic component in DF models is autocorrelated, the so-called Augmented Dickey–Fuller test is constructed (Dickey & Fuller, 1981). Model (3) is then transformed as

7 $$\begin{aligned} y_{t}=\phi _{1} y_{t-1}+\sum _{i=1}^{p-1} \gamma _{i} \varDelta y_{t-i}+\varepsilon _{t}, \end{aligned}$$

and the following equation is used to calculate the test statistic of the ADF test:

8 $$\begin{aligned} \varDelta y_{t}=\left( \phi _{1}-1\right) y_{t-1}+\sum _{i=1}^{p-1} \gamma _{i} \varDelta y_{t-i}+\varepsilon _{t}. \end{aligned}$$

#### Phillips–Perron test (PP test)

There is typically a problem selecting lag *p* in the regression model when unit-root testing time-series generated by a process with the autocorrelated and heteroscedastic non-systematic component. Instead of using appropriate autocorrelation models to describe the autocorrelation structure of the generating process, Phillips and Perron (1988) employed the usual Dickey–Fuller test with non-parametrically adjusted test statistics. This test is also founded on the models (3) and with the variation being that the linear trend in the last model is replaced by a time variable that is centred.

#### KPSS test

The null hypothesis states that the time-series $y_t$ is integrated of order one, *I*(1), as tested by all of the following tests. The KPSS test describes the opposite case, namely testing the null hypothesis that the time-series $y_t$ is *I*(0) (Kwiatkowski et al., 1992).

The Kwiatkowski–Phillips–Schmidt–Shin (KPSS) test determines whether a time-series is stationary or non-stationary around a mean or linear trend due to a unit root. A stationary time-series has statistical qualities that remain constant across time, such as the mean and variance.

The KPSS test is based on linear regression. It breaks up a series into three parts: a deterministic trend ($\beta _{t}$), a random walk ($r_t$), and a stationary error ($\varepsilon _{t}$), with the regression equation

9 $$\begin{aligned} x_{t}=r_{t}+\beta _{t}+\varepsilon _{1}. \end{aligned}$$

If the data are stationary, it will have a fixed element for an intercept or the series will be stationary around a fixed level (Wang, 2006).

The test uses ordinary least squares (OLS) find the equation, which differs slightly depending on whether you want to test for level stationarity or trend stationarity (Kočenda & Černỳ, 2015). A simplified version, without the time trend component, is used to test level stationarity.

Data are normally log-transformed before running the KPSS test, to turn any exponential trends into linear ones.

### Johansen and Juselius cointegration test

Johansen procedures (Johansen & Juselius, 1990) use two tests to determine the number of cointegration vectors: the Maximum Eigenvalue test and the Trace test. The Maximum Eigenvalue statistic tests the null hypothesis of *r* cointegrating relations against the alternative of $r+1$ cointegrating relations for $r = 0, 1, 2 \ldots n-1$. This test statistics are computed as

10 $$\begin{aligned} L R_{\max }(r / n+1)=-T * \log (1-{\hat{\lambda }}), \end{aligned}$$

where $\lambda$ is the maximum eigenvalue and *T* is the sample size. Trace statistics investigate the null hypothesis of *r* cointegrating relations against the alternative of *n* cointegrating relations, where *n* is the number of variables in the system for $r = 0, 1, 2 \ldots n-1$. Its equation is computed according to the following formula:

11 $$\begin{aligned} L R_{{\mathrm{tr}}}(r / n)=-T * \sum _{i=r+1}^{n} \log (1-{\hat{\lambda }}_{i}). \end{aligned}$$

In some cases, trace and maximum eigenvalue statistics may yield different results, and Alexander (2001) indicates that in this case, the results of trace test should be preferred.

### Granger causality test

Initially, we will assume that all variables are stationary. If the original variables have unit roots, then we assume that differences have been taken, such that the model includes the changes in the original variables (which do not have unit roots).

When we investigated Granger causality between *X* and *Y*, we began with an Autoregressive Distributed Lag Model $\hbox {ADL}(p, q)$ model for *Y* as the dependent variable.

An $\hbox {ADL}(p,q)$ model assumes that a time-series $Y_{t}$ can be represented by a linear function of *p* of its lagged values and *q* lags of another time-series $X_{t}$

12 $$\begin{aligned} Y_{t}= & {} \beta _{0}+\beta _{1} Y_{t-1}+\beta _{2} Y_{t-2}+\cdots +\beta _{p} Y_{t-p} \nonumber \\&+\delta _{1} X_{t-1}+\delta _{2} X_{t-2}+\cdots +\delta _{q} X_{t-q}+u_{t} \end{aligned}$$

is an autoregressive distributed lag model with *p* lags of $Y_t$ and *q* lags of $X_t$, where

13 $$\begin{aligned} E\left( u_{t} \mid Y_{t-1}, Y_{t-2}, \ldots , X_{t-1}, X_{t-2}, \ldots \right) =0. \end{aligned}$$

We used this model to investigate if *X* Granger caused *Y*. We then went on to consider causality in the other direction, which involved switching the roles of *X* and *Y* in the ADL. In particular, *X* became the dependent variable. We can write the two equations as follows:

14 $$\begin{aligned}&Y_{t}=\alpha _{1}+\delta _{1} t+\phi _{11} Y_{t-1}+\cdots +\phi _{1 p} Y_{t-p}+\beta _{11} X_{t-1}+\cdots +\beta _{1 q} X_{t-q}+\epsilon _{1 t}\nonumber \\&X_{t}=\alpha _{2}+\delta _{2} t+\phi _{21} Y_{t-1}+\cdots +\phi _{2 p} Y_{t-p}+\beta _{21} X_{t-1}+\cdots +\beta _{2 q} X_{t-q}+\epsilon _{2 t}.\nonumber \\ \end{aligned}$$

The first of these equations tests whether *X* Granger causes *Y*; the second, whether *Y* Granger causes *X*. Note that now the coefficients have subscripts indicating which equation they are in. The errors now have subscripts to denote the fact that they will be different in the two equations.

### Cointegration and VECM

When cointegration is identified between series, it is known that there exists a long-term equilibrium relationship between them, so we use VECM to evaluate the cointegrated series’ short-run features. If there is no cointegration, we skip VECM and go straight to Granger causality tests to determine the causal associations between variables. The regression equation form for VECM is as follows:

15 $$\begin{aligned}&\varDelta Y_{t}=\alpha _{1}+p_{1} e_{1}+\sum _{i=0}^{n} \beta _{i} \varDelta Y_{t-i}+\sum _{i=0}^{n} \delta _{i} \varDelta X_{t-i}+\sum _{i=0}^{n} \gamma _{i} Z_{t-i}\nonumber \\&\varDelta X_{t}=\alpha _{2}+p_{2} e_{t-1}+\sum _{i=0}^{n} \beta _{i} Y_{t-i}+\sum _{i=0}^{n} \delta _{i} \varDelta X_{t-i}+\sum _{i=0}^{n} \gamma _{i} Z_{t-i}. \end{aligned}$$

All variables are transformed in their log forms to mitigate inconsistency in the data and ease interpretation of the results via elasticities, the following is the empirical specifications for the model can be quantified as:

16 $$\begin{aligned} \hbox {COVID}19_{t}= & {} \beta _{0}{+} \beta _{1} [\hbox {O}_3]_{t-1}{+} \beta _{2}[\hbox {CO}]_{t-1}+ \beta _{3}[\hbox {SO}_2]_{t-1} {+} \beta _{4}[\hbox {PM}_{10}]_{t-1}{+} \beta _{5}[\hbox {SO}_2]_{t-1}\nonumber \\&+ \beta _{6}[\hbox {NO}_2]_{t-1} + \beta _{7}[\hbox {Temp}]_{t-1} + \beta _{8}[\hbox {RH}]_{t-1} + \beta _{9}[\hbox {WS}]_{t-1} +\varepsilon _{t}, \end{aligned}$$

where COVID-19 admitted cases are the dependent variable, while $\hbox {O}_3$, $\hbox {SO}_2$, $\hbox {NO}_2$, $\hbox {CO}$, $\hbox {PM}_{10}$, temperature, relative humidity (RH), and wind speed (WS) are the explanatory variables in days *t*, $\varepsilon _{t}$ is the error term, and $\beta _{0}, \beta _{1}, \beta _{2}, \beta _{3}, \beta _{4}, \beta _{5},\beta _{6},\beta _{7},\beta _{8}\ \hbox {and}\ \beta _{9}$ are the elasticities to be estimated.

However, a linear function can be used to express the relationship between number of COVID-19 cases and air pollution in Kuwait, as showed in the following expression:

17 $$\begin{aligned} {\hbox {COVID}19}_{t}=f\left( {\hbox {O}_3}_{t}, {\hbox {CO}}_{t}, {\hbox {PM}_{10}}_{t}, {\hbox {SO}_2}_{t}, {\hbox {NO}_2}_{t}, \hbox {Temp}, \hbox {RH}, \hbox {WS}\right) . \end{aligned}$$

Vector autoregression (VAR) model is first considered in the study following the work of Asumadu-Sarkodie and Owusu (2016), Chang (2010) and Gul et al. (2015).

Which can be expressed as

18 $$\begin{aligned} Y_{t}=\delta +A_{1} Y_{t-1}+\cdots +A_{p} Y_{t-p}+\varepsilon _{t}. \end{aligned}$$

The corresponding VEC model can be expressed as

19 $$\begin{aligned} \varDelta y_{t}= & {} \beta _{y_{0}}+\beta _{y_{1}} \varDelta y_{t-1}+\cdots +\beta _{y_{p}} \varDelta y_{t-p}+\gamma _{y_{1}} \varDelta x_{t-1} \nonumber \\&+\cdots +\gamma _{y_{p}} \varDelta x_{t-p}-\lambda _{y}\left( y_{t-1}-\alpha _{0}-\alpha _{1} x_{t-1}\right) +v_{t}^{y}\end{aligned}$$

20 $$\begin{aligned} \varDelta x_{t}= & {} \beta _{x_{0}}+\beta _{x_{1}} \varDelta y_{t-1}+\cdots +\beta _{x_{p}} \varDelta y_{t-p}+\gamma _{x_{1}} \varDelta x_{t-1} \nonumber \\&+\cdots +\gamma _{x_{p}} \varDelta x_{t-p}-\lambda _{x}\left( y_{t-1}-\alpha _{0}-\alpha _{1} x_{t-1}\right) +v_{t}^{x}, \end{aligned}$$

where $y_{t}=\alpha _{0}+\alpha _{1} x_{t}$ is the long-run cointegrating relation existing between two variables of interest, and $\lambda _{y}$ and $\lambda _{x}$ are the error correction parameters measuring the reaction of *y* and *x* towards the deviations from long-run equilibrium

Long run and cointegration between variables can be examined via several methods [e.g., Engle & Granger 1987, Johansen’s method (Johansen, 1995), Dynamic Ordinary Least Squares (DOLS), Fully Modified Ordinary Least Squares (FMOLS), and VEC models] for which variables need to be either *I*(1) or there needs to be prior knowledge and specification of variables as *I*(0) and *I*(1). An ARDL model can be used to estimate cointegration among variables at either *I*(0) or *I*(1) without the need to pre-specify which variables are *I*(0) or *I*(1) (Pesaran et al., 1995). Furthermore, an ARDL model does not require symmetry lag lengths and can have different number of lag terms unlike other cointegration estimation methods (Pesaran et al., 1995). In the present study, the long-run equilibrium relationship between number of COVID-19 cases and the independent variables ($\hbox {SO}_2$ and $\hbox {O}_3$) was estimated using the VEC model of cointegration.

## Results and discussion

### The descriptive statistics

Table 1 shows the descriptive statistics for the air pollutant variables. The mean value corresponding to $\hbox {O}_3$, $\hbox {CO}$, $\hbox {PM}_{10}$, $\hbox {SO}_2$ and $\hbox {NO}_2$ was $24.82\pm 7.20$, $9.11\pm 3.61$, $79.51\pm 24.45$, $11.24\pm 5.21$, and $26.72\pm 13.00$, respectively. It is also evident that except $\hbox {O}_3$, all the pollutants were positively skewed, i.e., mean values of these pollutants were high as compared to the median value. Moreover, Shapiro–Wilk test shows that the distributions of the variables were significantly differ from normal distribution. Therefore, log-transformation will be applied on the variables to convert the distribution of the variable to be normal distribution, before performing any further analysis. Minimum, maximum and percentile values of the pollutants are also shown in Table 1.

**Table 1:** Descriptive statistics

	$\hbox {O}_3$	$\hbox {CO}$	$\hbox {PM}_{10}$	$\hbox {SO}_2$	$\hbox {NO}_2$
Mean	24.818	9.112	79.511	11.239	26.719
Median	25.398	8.184	76.334	10.001	24.529
Std. deviation	7.202	3.611	24.452	5.214	12.996
Skewness	0.026	1.969	2.542	1.383	0.514
Kurtosis	0.514	6.324	11.931	2.325	0.521
Shapiro–Wilk (SW)	0.991	0.844	0.813	0.895	0.959
*P*-value of (SW)	0.066	0.001	0.001	0.001	0.001
Minimum	9.250	3.791	35.357	3.802	5.154
Maximum	42.226	28.445	234.057	34.443	64.515
25th percentile	19.249	6.618	67.104	7.523	15.502
50th percentile	25.398	8.184	76.334	10.001	24.529
75th percentile	29.755	10.936	87.004	13.906	35.561

Descriptive statistics for daily climatology variable (RH, Temp, WD, and WS), COVID-19 cases, and COVID-19 deaths are shown in Table 2. The mean value for RH, Temp, WD, and WS is 35.51 (SD = 20.06), 30.26 (SD = 7.96), 206.138 (SD = 54.48), and 2.18 (SD = 0.66), respectively. Moreover, on an average, 506 cases of COVID-19 and 3 deaths due to COVID-19 were reported in the study period. Results of Shapiro–Wilk test shows that the distributions of the climatology parameters, COVID-19 cases, and COVID-19 deaths were different from the normal distribution. Therefore, log-transformation will be applied on the variables to convert the distribution of the variables to be normal. The value of other test statistics, i.e., median, skewness, kurtosis, minimum, maximum, and percentile for each variable is also shown in Table 2.

**Table 2:** Descriptive statistics

	RH	Temp	WD	WS	COVID-19 cases	Death cases
Mean	35.506	30.255	206.138	2.183	506.436	3.145
Median	28.010	31.141	199.509	2.020	554.500	3.000
Std. deviation	20.062	7.956	54.481	0.662	276.988	2.524
Skewness	0.793	0.371	0.123	0.786	0.290	0.918
Kurtosis	0.367	1.069	1.202	0.106	0.902	0.513
Shapiro–Wilk (SW)	0.897	0.935	0.948	0.948	0.956	0.915
*p*-value of (SW)	0.001	0.001	0.001	0.001	0.001	0.001
Minimum	11.619	12.199	89.663	0.938	1.000	0.000
Maximum	91.534	43.150	297.845	4.271	1073.000	11.000
25th percentile	18.564	24.714	160.862	1.670	278.000	1.000
50th percentile	28.010	31.141	199.509	2.020	554.500	3.000
75th percentile	51.462	37.501	257.648	2.606	711.750	4.000

*RH* relative humidity, *Temp.* temperature in Celsius, *WD* wind direction, *WS* wind speed, *COVID-19 cases* daily reported cases from Kuwait ministry of health

### Correlation analysis

Table 3 present results of the correlation analysis for air pollutants, climatology parameters, and COVID-19 cases. A strong significant positive correlation was observed between temperature and COVID-19 cases ($r_{\mathrm{p}}=0.61$), indicated that as the value of temperature increases, COVID-19 cases also increase, whereas a negative significant correlation was observed between RH and COVID-19 cases ($r_{\mathrm{p}}= -0.49$), indicated that as the value of relative humidity (RH) increases, COVID-19 cases decrease. Moreover, a small effect of $\hbox {O}_3$ ($r_{\mathrm{p}}=0.25$), $\hbox {CO}$ ($r_{\mathrm{p}}=0.24$), $\hbox {PM}_{10}$ ($r_{\mathrm{p}}=0.18$) and $\hbox {NO}_2$ ($r_{\mathrm{p}}=0.22)$ was also observed on COVID-19 cases.

**Table 3:** Pearson’s correlations

Variable	$\hbox {O}_3$	$\hbox {CO}$	$\hbox {PM}_{10}$	$\hbox {SO}_2$	$\hbox {NO}_2$	RH	Temp	WD	WS
1. $\hbox {O}_3$	–								
2. $\hbox {CO}$	$-$0.419***	–							
3. $\hbox {PM}_{10}$	0.124*	0.080	–						
4. $\hbox {SO}_2$	0.033	0.008	$-$0.044	–					
5. $\hbox {NO}_2$	$-$0.452***	0.614***	$-$0.002	0.132*	–				
6. RH	$-$0.491***	0.228***	$-$0.228***	$-$0.391***	$-$0.027	–			
7. Temp	0.461***	0.009	0.292***	0.124*	0.010	$-$0.753***	–		
8. WD	0.026	$-$0.233***	0.085	0.272***	$-$0.035	$-$0.411***	0.134*	–	
9. WS	0.350***	$-$0.333***	0.317***	$-$0.088	$-$0.492***	$-$0.139*	0.206***	0.317***	–
10. COVID-19 cases	0.248***	0.235***	0.182**	0.114	0.222***	$-$0.487***	0.608***	0.157**	$-$0.025

*$$p < 0.05,$$ **$$p<0.01,$$ ***$$p <0.001$$

### Results of Granger causality test

Granger causality test has been conducted to check if the series of independent variables is useful for making prediction or not. Results of the Granger causality test have been shown in the following subsection.

#### Results of the unit-root tests

Unit-root tests for each variable were conducted before the main analysis to avoid spurious regression in time-series research (Mahadeva & Robinson, 2004). Some air pollutant and climatology parameters were significantly associated with COVID-19, so VECM analysis was done to assess the short- and long-term relationship of these variables. Since non-stationary series may also product spurious regression results for VECM (Asari et al., 2011; Latief et al., 2021), stationary tests on the time-series data were performed using the conventional Augmented Dickey–Fuller (ADF-GLS), and Phillips–Perron (PP) and KPSS Tests. ADF results are shown in Tables 4 and 5, and PP and KPSS tests are shown in Tables 6 and 7.

Tables 4 and 5 present the results of the ADF-GLS unit roots test in levels and first differences (results with constant are presented in Table 4, and results with constant and trend are presented in Table 5). The results of test confirm that all variables used in research (COVID-19 cases, $\hbox {O}_3$, $\hbox {SO}_2$, $\hbox {NO}_2$, $\hbox {CO}$, and $\hbox {PM}_{10}$) are integrated of first order *I*(1). The ADF-GLS test results for COVID-19 cases, $\hbox {CO}$, $\hbox {PM}_{10}$ and $\hbox {SO}_2$ where the null hypothesis of non-stationarity is rejected for levels (test with constant), what means that recreation COVID-19 cases because of air pollution ($\hbox {CO}$, $\hbox {PM}_{10}$ and $\hbox {SO}_2$) could be a stationary process. In addition to that, the ADF-GLS (test with constant and trend $(c+t)$) results for $\hbox {O}_3$, $\hbox {SO}_2$, $\hbox {NO}_2$, $\hbox {CO}$, and $\hbox {PM}_{10}$ where the null hypothesis of non-stationarity are rejected for levels, what means that recreation COVID-19 cases because of air pollution ($\hbox {O}_3$, $\hbox {SO}_2$, $\hbox {NO}_2$, $\hbox {CO}$, and $\hbox {PM}_{10}$) could be a stationary process over a trend. However, the KPSS test (where the null hypothesis of stationarity is rejected for levels) suggest the first order of integration (*I*(1)) for all variables except $\hbox {SO}_2$ (see Table 6). Because of the inconsistency in ADF test, we diced to take the KPSS test results under consideration. Hence, all variables except $\hbox {SO}_2$ in levels are *I*(1) variables, and then, the cointegration analysis were conducted in next step.

**Table 4:** ADF-GLS root tests with constant

	Level			First difference		
	Coefficient	*t*-ratio	*p*-value	Coefficient	*t*-ratio	*p*-value
Log($\hbox {O}_3$)	$-$0.0708452	$-$1.854	0.3548	$-$0.373708	$-$5.953	7.67e$-$09***
Log($\hbox {CO}$)	$-$0.151600	$-$3.869	0.0023***	$-$0.120178	$-$1.911	0.0570*
Log($\hbox {PM}_{10}$)	$-$0.401449	$-$5.633	8.68e$-$07***	$-$0.129774	$-$1.770	0.0778*
Log($\hbox {SO}_2$)	$-$0.235017	$-$4.358	0.0003***	$-$0.183925	$-$2.763	0.0061***
Log($\hbox {NO}_2$)	$-$0.0768789	$-$2.393	0.1436	$-$0.253951	$-$4.118	5.01e$-$05***
Log(COVID-19 cases)	$-$0.0460937	$-$2.932	0.0417**	$-$0.393289	$-$6.970	2.18e$-$11***

*Stationarity at 10% significance levels, **stationarity at 5% significance levels, ***stationarity at 1% significance levels

**Table 5:** ADF-GLS root tests with constant and trend

	Level			First difference		
	Coefficient	*t*-ratio	*p*-value	Coefficient	*t*-ratio	*p*-value
Log($\hbox {O}_3$)	$-$0.183827	$-$3.594	0.0303**	$-$0.305549	$-$4.686	4.31e$-$06***
Log($\hbox {CO}$)	$-$0.247380	$-$5.165	8.51e$-$05***	$-$0.0613829	$-$0.9560	0.3399
Log($\hbox {PM}_{10}$)	$-$0.427096	$-$5.832	2.93e$-$06***	$-$0.112507	$-$1.518	0.1300
Log($\hbox {SO}_2$)	$-$0.235853	$-$4.362	0.0025***	$-$0.183525	$-$2.752	0.0063***
Log($\hbox {NO}_2$)	$-$0.184001	$-$4.066	0.0070***	$-$0.187636	$-$2.938	0.0036***
Log(COVID19 cases)	$-$0.0382004	$-$2.171	0.5055	$-$0.403573	$-$7.036	1.47e$-$11***

*Stationarity at 10% significance levels, **stationarity at 5% significance levels, ***stationarity at 1% significance levels

**Table 6:** Unit-root tests using KPSS with constant and trend

	Level			First difference		
	Coefficient	*t*-ratio	*p*-value	Coefficient	*t*-ratio	*p*-value
Log($\hbox {O}_3$)	$-$0.00227119	$-$13.02	6.43e$-$31***	$-$8.82232e$-$05	$-$0.6322	0.5278
Log($\hbox {CO}$)	0.00210608	10.56	2.53e$-$22***	6.37E$-$05	0.4359	0.6632
Log($\hbox {PM}_{10}$)	$-$0.000566757	$-$3.161	0.0017***	9.78E$-$06	0.05286	0.9579
Log($\hbox {SO}_2$)	0.000286053	0.9786	0.3286	4.94E$-$05	0.2043	0.8383
Log($\hbox {NO}_2$)	0.00402837	14.63	8.82e$-$37***	7.88E$-$05	0.3921	0.6953
Log(COVID19 cases)	0.00685064	8.903	5.74e$-$17***	$-$0.000319900	$-$1.302	0.1940

*Stationarity at 10% significance levels, **stationarity at 5% significance levels, ***stationarity at 1% significance levels

**Table 7:** Phillips–Perron (PP) unit-root test constant and trend

	Constant			Trend		
	Coefficient	*t*-ratio	*p*-value	Coefficient	*t*-ratio	*p*-value
Log($\text {O}_3$)	0.80646	22.523	2e$-$16***	6.76E$-$01	15.73	2e$-$16***
$\varDelta$ Log($\hbox {O}_3$)	$-$0.301562	$-$5.398	1.4e$-$07***	$-$0.3029678	$-$5.417	1.27e$-$07***
Log($\text {CO}$)	0.32928	5.971	6.81e$-$09***	3.10E$-$01	5.567	5.87e$-$08***
$\varDelta$ Log($\hbox {CO}$)	$-$0.35475	$-$6.482	3.85e$-$10***	$-$3.55E$-$01	$-$6.471	4.13e$-$10***
Log($\hbox {PM}_{10}$)	0.49101	9.655	2e$-$16***	4.74E$-$01	9.203	2e$-$16***
$\varDelta$ Log($\hbox {PM}_{10}$)	$-$0.26979	$-$4.784	2.73e$-$06***	$-$2.70E$-$01	$-$4.776	2.84e$-$06***
Log($\hbox {SO}_2$)	0.66515	15.129	2e$-$16***	6.64E$-$01	15.066	2e$-$16***
$\varDelta$ Log($\hbox {SO}_2$)	$-$0.24294	$-$4.279	2.55e$-$05***	$-$2.43E$-$01	$-$4.274	2.61e$-$05***
Log($\hbox {NO}_2$)	0.8495	27.27	2e$-$16***	7.28E$-$01	18.49	2e$-$16***
$\varDelta$ Log($\hbox {NO}_2$)	$-$0.184247	$-$3.198	0.00153**	$-$1.85E$-$01	$-$3.202	0.00152**
Log(COVID19 cases)	0.9394	58.309	2e$-$16***	0.9354317	51.365	2e$-$16***
$\varDelta$ Log(COVID19 cases)	$-$0.29767	$-$5.327	1.99e$-$07***	$-$0.306341	$-$5.489	8.81e$-$08***

*Stationarity at 10% significance levels, **stationarity at 5% significance levels, ***stationarity at 1% significance levels

ADF root test with constant shows that the time-series for pollutants $\hbox {CO}$, $\hbox {PM}_{10}$, $\hbox {SO}_2$ and series of COVID-19 cases is stationary at 5%-level Dickey–Fuller criterion, whereas series of $\hbox {O}_3$ and $\hbox {NO}_2$ was stationary at first difference (Table 4). The results of ADF test with constant and trend demonstrate that the series of all the pollutants are stationary at level, whereas series of COVID-19 was stationary at first difference (Table 5). The results of PP unit-root test shows that the series of all the pollutant variables and COVID-19 cases is stationary at level as well as on the first difference (Table 7). The results of KPSS test shows that the series of all the pollutants (except $\hbox {SO}_2$) and COVID-19 cases are non-stationary at level, though all the series are found to be stationary at first difference (Table 6).

#### Estimation of VAR model

After checking the stationarity of the series, the next step is to determine the number of optimal lags. To choose the number of lags need to be included in the VAR model, VARselect function has been taken into consideration. This function calculates three different information criteria across a number of different lags (up to a maximum specified within the function) and chooses the lag that has the lowest information criteria for each of the three statistics. The asterisks symbol indicate the best values under the respective information criteria, AIC = Akaike criterion, BIC = Schwarz Bayesian criterion, and HQC = Hannan–Quinn criterion. Table 8 illustrates the results of lag order statistics. Akaike information criteria statistics suggests that the optimal lag order for the model is 3.

**Table 8:** Lag selection criterion VAR test using trend model with the endogenous series Log(COVID-19 Kuwait), Log($\text {O}_3$), Log($\text {SO}_2$), Log($\text {NO}_2$), Log($\text {CO}$), and Log($\text {PM}_{10}$) with weather factors (temperature, relative humidity, and wind speed)

Lags	Loglik	p(LR)	AIC	BIC	HQC
1	$-$1325.47546		10.322286	11.620908*	10.843401*
2	$-$1214.66729	0.00000	10.106285	12.467416	11.053767
3	$-$1130.15629	0.00000	10.080843*	13.504483	11.454692
4	$-$1067.99268	0.00141	10.217338	14.703487	12.017554
5	$-$1012.44854	0.01490	10.401801	15.950459	12.628384
6	$-$946.13449	0.00026	10.508221	17.119388	13.161171
7	$-$902.00189	0.27205	10.775376	18.449052	13.854693
8	$-$850.35647	0.04809	10.988090	19.724275	14.493774
9	$-$771.15278	0.00000	11.001107	20.799801	14.933158
10	$-$706.61080	0.00055	11.120368	21.981571	15.478786

*LogLik* log-likelihood, *p(LR)* likelihood ratio test (LR), *AIC* Akaike criterion, *BIC* Schwarz Bayesian criterion, *HQC* Hannan–Quinn criterion

*Stationarity at 5% significance levels,**stationarity at 1% significance levels

#### Johansen cointegration tests

The long-term relationship among variables was checked with the Johansen cointegration test (Johansen, 1995) by max-eigenvalue and trace methods (Table 9). Based on these results and a 5% significance level, we reject the null hypothesis of no cointegration ($r=0$, trace test = 252.42, $p=0.00$) and fail to reject the null hypothesis that there are one or two cointegrating equations in the multivariate model.

This reveals that there exists at least one level of cointegration equation, which indicated that the variables have long-term relationship. Furthermore, the results of cointegration test show that exist at most two level of cointegration ($r\le 2$, trace test = 134.04, $p=0.0127$) between the times series of Log(COVID-19 Kuwait), Log($\hbox {O}_3$), Log($\hbox {SO}_2$), Log($\hbox {NO}_2$), Log($\hbox {CO}$), and Log($\hbox {PM}_{10}$) (Table 9).

**Fig. 1:**
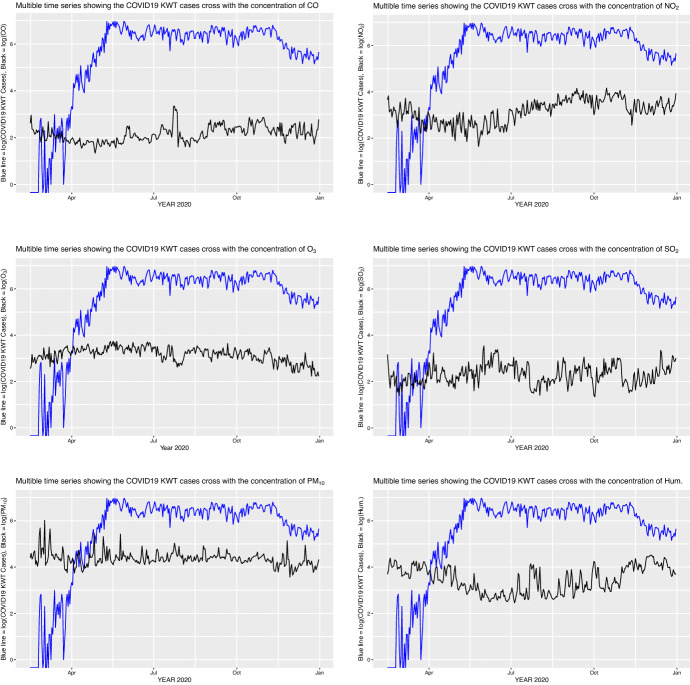
Daily time-series for Log COVID-19 Kuwait compared with Log($\hbox {O}_3$), Log($\hbox {SO}_2$), Log($\hbox {NO}_2$), Log($\hbox {CO}$), and Log($\hbox {PM}_10$)

Moreover, Engle and Granger (1987) suggested a two-step process to test the cointegration (an OLS regression and a unit-root test). According to Engle and Granger (1987), if a set of variables are cointegrated, then there exists a valid error correction representation of the data, and vice versa. Therefore, an analysis of OLS regression and error correction model has been performed to testing the cointegrating relationship $(r = 2)$ in a system of $k = 2$, *I*(1) variables. The results of cointegration regression analysis (Table 10) confirm that there is a long relationship between the series Log(COVID-19 Kuwait), Log($\hbox {O}_3$), Log($\hbox {SO}_2$), Log($\hbox {NO}_2$), Log($\hbox {CO}$), and Log($\hbox {PM}_{10}$). Figure 1 shows the simultaneous variation of Log(COVID19) with Log($O_3$) and Log($\hbox {SO}_2$).

**Table 9:** Johansen test for selecting the number of cointegration that reflect linear combination of underlying series to form a stationary series for Log(COVID-19 Kuwait), Log($\hbox {O}_3$), Log($\hbox {SO}_2$), Log($\hbox {NO}_2$), Log($\hbox {CO}$), and Log($\hbox {PM}_{10}$) with weather factors (temperature, relative humidity and wind speed)

Rank	Eigenvalue	Trace test	*p*-value	Lmax test	*p*-value
$r = 0$	0.19194	252.42	0.0000	62.016	0.0169
$r\le 1$	0.17608	190.40	0.0003	56.361	0.0143
$r\le 2$*	0.14023	134.04	0.0127	43.969	0.0335
$r\le 3$	0.10147	90.070	0.1142	31.136	0.3658
$r\le 4$	0.072278	58.934	0.2709	21.832	0.6292
$r\le 5$	0.053531	37.102	0.3469	16.010	0.6704

**Table 10:** Cointegration regression for the times series of Log(COVID-19 Kuwait), Log($\hbox {O}_3$), Log($\hbox {SO}_2$), Log($\hbox {NO}_2$), Log($\hbox {CO}$), and Log($\hbox {PM}_{10}$) with weather factors (temperature, relative humidity, and wind speed) using the model with constant and trend term

	Coefficient	Std. error	*t*-ratio	*p*-value
Const	1.86215	0.982174	1.896	0.0590
Log($\hbox {O}_3$)	0.515990	0.161498	3.195	0.001**
Log($\hbox {CO}$)	0.218875	0.158709	1.379	0.1689
Log($\hbox {PM}_{10}$)	0.383031	0.151655	2.526	0.0121*
Log($\hbox {SO}_2$)	$-$0.00739348	0.101779	$-$0.07264	0.9421
Log($\hbox {NO}_2$)	$-$1.21305	0.114179	$-$10.62	0.001 **
Log($\hbox {RH}$)	$-$0.00947287	0.00369657	$-$2.563	0.0109*
Log($\hbox {Temp}$)	0.102469	0.00816087	12.56	0.001 **
Log($\hbox {WS}$)	$-$0.440439	0.0673461	$-$6.540	0.001 **
Time	0.0115185	0.000844428	13.64	0.001**

Dependent is Log(COVID-19 Kuwait), *stationarity at 5% significance levels, **stationarity at 1% significance levels. *R*-squared = 0.791, adjusted *R*-squared = 0.785, Akaike criterion = 537.269, Durbin–Watson = 0.797

### Determination of optimal VECM

VECM analysis with restricted constant and restricted trend results is shown in Table 11. Past COVID-19 cases, $\hbox {NO}_2$, $\hbox {SO}_2$, $\hbox {Temp}$, $\hbox {RH}$ and $\hbox {WS}$, were significantly associated with future COVID-19 cases in Kuwait. The Error Correction Model (ECT) shows how fast variables return to long-run equilibrium when a cointegration relationship exists. EC1 is negative and significant indicating a long-run causality of future COVID-19 cases with past COVID-19 cases. The error correction term explains 5.102% disequilibrium of COVID-19 cases in Kuwait compared to other variables. The speed of adjustment at 5.102% towards the long run is also explained at the 1% significance level.

**Table 11:** VECM equation that predicting COVID19 cases with restricted trend, lag order = 3, and cointegration rank order = 2

	Coefficient	Std. error	*t*-ratio	*p*-value
Const	0.345343	0.487003	0.7091	0.4789
$\varDelta$ log(COVID19)$_{{\mathrm{lag}}=1}$	$-$0.312405	0.0623067	$-$5.014	0.0001***
$\varDelta$ log(COVID19)$_{{\mathrm{lag}}=2}$	$-$0.143473	0.0579692	$-$2.475	0.0139**
$\varDelta$ log${(\hbox {O}_{3})}_{{\mathrm{lag}}=1}$	$-$0.122409	0.114960	$-$1.065	0.2879
$\varDelta$ log${(\hbox {O}_{3})}_{{\mathrm{lag}}=2}$	0.007500	0.112985	0.06638	0.9471
$\varDelta$ log$(\hbox {CO})_{{\mathrm{lag}}=1}$	$-$0.113318	0.124636	$-$0.9092	0.3641
$\varDelta$ log$(\hbox {CO})_{{\mathrm{lag}}=2}$	$-$0.026752	0.120734	$-$0.2216	0.8248
$\varDelta$ log$(\hbox {PM}_{10})_{{\mathrm{lag}}=1}$	$-$0.178503	0.111194	$-$1.605	0.1096
$\varDelta$ log$(\hbox {PM}_{10})_{{\mathrm{lag}}=2}$	$-$0.141589	0.0928460	$-$1.525	0.1284
$\varDelta$ log${(\hbox {SO}_{2})}_{{\mathrm{lag}}=1}$	$-$0.152499	0.0670539	$-$2.274	0.0237**
$\varDelta$ log${(\hbox {SO}_{2})}_{{\mathrm{lag}}=2}$	0.045896	0.0670338	0.6847	0.4941
$\varDelta$ log${(\hbox {NO}_{2})}_{{\mathrm{lag}}=1}$	0.348294	0.109700	3.175	0.0017***
$\varDelta$ log$(\hbox {NO}_{2})_{{\mathrm{lag}}=2}$	0.147046	0.0987793	1.489	0.1377
$\varDelta$ log$(\hbox {Temp})_{{\mathrm{lag}}=1}$	0.053360	0.0166922	3.197	0.0016***
$\varDelta$ log$(\hbox {Temp})_{{\mathrm{lag}}=2}$	0.001178	0.0168422	0.06994	0.9443
$\varDelta$ log$(\hbox {RH})_{{\mathrm{lag}}=1}$	0.006932	0.00281235	2.465	0.0143**
$\varDelta$ log$(\hbox {RH})_{{\mathrm{lag}}=2}$	$-$0.002858	0.00296389	$-$0.9642	0.3358
$\varDelta$ log$(\hbox {WS})_{{\mathrm{lag}}=1}$	0.051015	0.0524016	0.9735	0.3312
$\varDelta$ log$(\hbox {WS})_{\mathrm{lag}=2}$	0.118484	0.0467846	2.533	0.0119**
EC1	$-$0.056697	0.027537	$-$2.059	0.0405**
EC2	$-$0.014895	0.013930	$-$1.069	0.2859
Mean dependent var	0.012207	SD dependent var		0.356198
Residual sum of squares	27.82260	SS.E. of regression		0.320416
*R*-squared	0.249	Adjusted *R*-squared		0.191
rho	$-$0.0381	Durbin–Watson		2.0428

*, **, *** imply that we can reject the null hypothesis at 10%, 5% and 1% significant levels, respectively

**Fig. 2:**
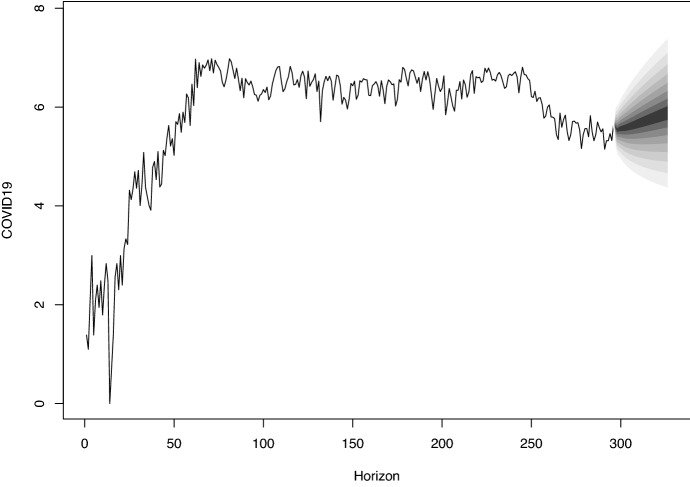
Forecasting COVID-19 cases using VECM

Figure 2 shows the future trend of COVID-19 using VECM. From Fig. 2, it can be observed that the forecasted value shows a linear trend and the predicted value lies within 95% the confidence interval.

## Conclusion and recommendations

The primary goal of the current study is to look into the association between changes in daily admitted COVID-19 cases and air pollution levels during the Corona pandemic from March to December 2020. Medical analysts, policymakers, environmental decision-makers, and anyone interested in measuring the causality relationship between daily admitted COVID-19 cases and air pollution, such as the World Health Organization (WHO), through a series of policies for this situation. Based on a descriptive analysis of the variables, the association between air pollutants ($\hbox {O}_3$, $\hbox {SO}_2$, $\hbox {NO}_2$, $\hbox {CO}$, and $\hbox {PM}_{10}$) and daily admitted COVID-19 patients has been established; this is consistent with the literature reviewed. This research used the vector error corrected model (VECM) with cointegration technique to look at the long- and short-run association between the effect of air pollution ($\hbox {O}_3$, $\hbox {SO}_2$, $\hbox {NO}_2$, $\hbox {CO}$, and $\hbox {PM}_{10}$) and the daily admitted COVID-19 cases. We discovered that for COVID-19 patients, a greater AQI was linked to a higher number of hospitalizations.

The outcomes for COVID-19 patients showed that increasing the air quality index has a positive and significant effect on increasing the admitted number of COVID-19 patients. The lags of the dependent variable are significant until the second lags, implying that increasing the air quality index, particularly for $\hbox {O}_3$ and $\hbox {SO}_2$, affects increasing the number of COVID-19 patients with long delays. The model for correcting errors revealed that about 7% of the short-term imbalance is rectified in the event of a shock to achieve long-term balance in just one day. In the long run, boosting the air quality index for $\hbox {O}_3$ and $\hbox {SO}_2$ has been successful in increasing the admitted number of COVID-19 patients.

The coefficient of the air pollution index is positive and substantial for COVID-19 patients who are hospitalized. This suggests that raising the air quality index ($\hbox {O}_3$ and $\hbox {SO}_2$) can increase the number of COVID-19 patients that are admitted to the hospital. This is in line with a study that found a link between COVID-19 infection and air pollution, which has a significant impact on infection and mortality rates (Frontera et al., 2021). Using a time-series method, another study in Chile, Dales et al. (2021), found a significant association between acute IQR increases in $\hbox {CO}$, $\hbox {NO}_2$, and $\hbox {PM}_{2.5}$ and increases of around 6% in daily COVID-19 associated deaths.

When the health variables were examined, it was shown that the majority of the people infected with COVID-19 were already exposed to air pollution, because Kuwait’s regions have significant pollution rates. The biggest cause of pollution has been air pollutants emitted by cars and businesses (Hamoda et al., 2020). COVID-19 impacts the human respiratory system, and people who are already susceptible to the respiratory disease have a propensity to be affected by the pandemic (Ghanim, 2021).

COVID-19’s lockdown analyzed human activities, mostly involving vehicle usage and public transportation, as well as industrial processes (Gautam, 2020; Pata, 2020; Shehzad et al., 2020). The importance of air pollution and COVID-19 has been demonstrated in numerous studies. The spread of COVID-19 has been found predominant through airborne bio-aerosol droplets together with various aspects of urban air pollution (Fareed et al., 2020). Past exposure to air pollution has led to an increase in the cases of COVID-19. The ability to transfer these viruses is demonstrated by air pollution. We approximated the error correction model based on the VECM procedure to obtain short-term coefficients after investigating the long-term findings. The results show that while $\hbox {O}_3$ and $\hbox {SO}_2$ have an increasing short-term effect, they have a long-term positive effect on the daily admitted COVID-19 case. The error correction term (ECT) is statistically significant and has a negative value, indicating that a deviation from the long-term equilibrium will be repaired. The findings show that the short-term coefficients of $\hbox {O}_3$ and $\hbox {SO}_2$ are lower than the long-term coefficients.

Our research has several limitations. We have to revert to the air quality index as a measure of air pollution level due to inadequate reporting on certain pollutants. This, however, may obscure the impact of certain contaminants on the number of hospitalizations. Furthermore, because our estimates focused on a single link between factors, any ascribed cost estimation should be cautiously approached. Other aspects, such as humidity, wind speed, and seasonality level, may need to be adjusted in the model (winter, autumn, spring, and summer). However, because their data were not available or valid in this study, we did not alter them.

Other time-series methods, such as the vector autoregression (VAR) model, which is one of the most effective, flexible, and user-friendly models for multivariate time-series analysis, could be recommended for future investigations. The basic model for studying a stationary time-series in terms of two polynomials is the autoregressive-moving average (ARMA) process. Other multivariate time-series analysis techniques include Vector Autoregression Moving-Average (VARMA), VARMAX (VARMAX with Exogenous Regressors), and Holt Winter’s Exponential Smoothing (HWES). A spatial multivariate time-series approach could be used to assess the distance between a job or a living area and a pollution source. Furthermore, taking critical key elements like wind speed and air humidity into account can help to minimize the disruption of damaged components.

## Data Availability

Data are provided for the reviewers as per need.

## Declarations

### Code availability

R-code is provided for the reviewers as per need.

## References

[CR1] Alexander, C. (2001). *Market Models. A Guide to Financial Data analysis*. Wiley, Chichester.

[CR2] Al Mulla A, Fanous N, Seidenberg AB, Rees VW (2015). Secondhand smoke emission levels in waterpipe cafes in Doha, Qatar. Tobacco Control.

[CR3] Amoatey P, Omidvarborna H, Baawain M (2018). The modeling and health risk assessment of PM$$_{2.5}$$ from Tema Oil Refinery. Human and Ecological Risk Assessment: An International Journal.

[CR4] Argyropoulos, C. D., Abraham, M., Hassan, H., Ashraf, A., Fthenou, E., Sadoun, E., & Kakosimos, K. (2016). Modeling of PM10 and PM2. 5 building infiltration during a dust event in Doha, Qatar. In *Proceedings of 2nd international conference on atmospheric dust-DUST2016, Castellaneta Marina-Taranto, Italy*.

[CR5] Asari F, Baharuddin NS, Jusoh N, Mohamad Z, Shamsudin N, Jusoff K (2011). A vector error correction model (VECM) approach in explaining the relationship between interest rate and inflation towards exchange rate volatility in Malaysia. World Applied Sciences Journal.

[CR6] Asumadu-Sarkodie S, Owusu PA (2016). The potential and economic viability of solar photovoltaic power in Ghana. Energy Sources, Part A: Recovery, Utilization, and Environmental Effects.

[CR7] Azlina A, Law SH, Mustapha NHN (2014). Dynamic linkages among transport energy consumption, income and CO$$_{2}$$ emission in Malaysia. Energy Policy.

[CR8] Bashir MF, Benghoul M, Numan U, Shakoor A, Komal B, Bashir MA, Bashir M, Tan D (2020). Environmental pollution and COVID-19 outbreak: Insights from Germany. Air Quality, Atmosphere & Health.

[CR9] Bashir MF, Ma B, Komal B, Bashir MA, Tan D, Bashir M (2020). Correlation between climate indicators and COVID-19 pandemic in New York, USA. Science of the Total Environment.

[CR10] Benvenuto D, Giovanetti M, Vassallo L, Angeletti S, Ciccozzi M (2020). Application of the ARIMA model on the COVID-2019 epidemic dataset. Data in Brief.

[CR11] Box, G. E. P., & Jenkins, G. M. (1970). *Time Series Analysis: Forecasting and Control*. Holden-Day, San Francisco.

[CR12] Briz-Redón Á, Serrano-Aroca Á (2020). A spatio-temporal analysis for exploring the effect of temperature on COVID-19 early evolution in Spain. Science of the Total Environment.

[CR13] Capan M, Hoover S, Jackson EV, Paul D, Locke R (2016). Time series analysis for forecasting hospital census: Application to the neonatal intensive care unit. Applied Clinical Informatics.

[CR14] Casanova LM, Jeon S, Rutala WA, Weber DJ, Sobsey MD (2010). Effects of air temperature and relative humidity on coronavirus survival on surfaces. Applied and Environmental Microbiology.

[CR15] Chang CC (2010). A multivariate causality test of carbon dioxide emissions, energy consumption and economic growth in China. Applied Energy.

[CR16] Cui Y, Zhang ZF, Froines J, Zhao J, Wang H, Yu SZ, Detels R (2003). Air pollution and case fatality of SARS in the People’s Republic of China: An ecologic study. Environmental Health.

[CR17] Dales R , Blanco-Vidal C, Romero-Meza R, Schoen S, Lukina A, Cakmak S (2021). The association between air pollution and COVID-19 related mortality in Santiago, Chile: A daily time series analysis. Environmental Research.

[CR18] Dickey, D. A. (1976). Estimation and Hypothesis Testing in Nonstationary Time Series. Ph.D. Dissertation, Iowa State University, Ames.

[CR19] Dickey DA, Fuller WA (1979). Distribution of the estimators for autoregressive time series with a unit root. Journal of the American Statistical Association.

[CR20] Dickey, D. A., & Fuller, W. A. (1981). Likelihood Ratio Statistics for Autoregressive Time Series with a Unit Root. *Econometrica,**49*(4), 1057–1072. 10.2307/1912517.

[CR21] Distante C, Piscitelli P, Miani A (2020). COVID-19 outbreak progression in Italian regions: Approaching the peak by the end of March in northern Italy and first week of April in southern Italy. International Journal of Environmental Research and Public Health.

[CR22] Domingo JL, Marqués M, Rovira J (2020). Influence of airborne transmission of SARS-CoV-2 on COVID-19 pandemic. A review. Environmental Research.

[CR23] Du W, Li X, Chen Y, Shen G (2018). Household air pollution and personal exposure to air pollutants in rural China—A review. Environmental Pollution.

[CR24] Earnest A, Chen MI, Ng D, Sin LY (2005). Using autoregressive integrated moving average (ARIMA) models to predict and monitor the number of beds occupied during a SARS outbreak in a tertiary hospital in Singapore. BMC Health Services Research.

[CR25] Engle, R. F., & Granger, C. W. J. (1987). Co-integration and error correction: representation, estimation, and testing. *Econometrica,**55*(2), 251–276. 10.2307/1913236.

[CR26] Fareed Z, Iqbal N, Shahzad F, Shah SGM, Zulfiqar B, Shahzad K, Hashmi SH, Shahzad U (2020). Co-variance nexus between COVID-19 mortality, humidity, and air quality index in Wuhan, China: New insights from partial and multiple wavelet coherence. Air Quality, Atmosphere & Health.

[CR27] Frontera JA, Sabadia S, Lalchan R, Fang T, Flusty B, Millar-Vernetti P, Snyder T, Berger S, Yang D, Granger A (2021). A prospective study of neurologic disorders in hospitalized patients with COVID-19 in New York City. Neurology.

[CR28] Fuller, W. A. (1976). *Introduction to statistical time series*. Wiley.

[CR29] Gautam S (2020). COVID-19: Air pollution remains low as people stay at home. Air Quality, Atmosphere & Health.

[CR30] Ghanim, A. A. J. (2022). Analyzing the severity of coronavirus infections in relation to air pollution: evidence-based study from Saudi Arabia. *Environmental Science and Pollution Research International,**29*(4), 6267–6277. 10.1007/s11356-021-15507-9. 10.1007/s11356-021-15507-9 PMC8390106 34448138

[CR31] Gul S, Zou X, Hassan CH, Azam M, Zaman K (2015). Causal nexus between energy consumption and carbon dioxide emission for Malaysia using maximum entropy bootstrap approach. Environmental Science and Pollution Research.

[CR32] Gupta S, Raghuwanshi GS, Chanda A (2020). Effect of weather on COVID-19 spread in the US: A prediction model for India in 2020. Science of the Total Environment.

[CR33] Hamoda, M. F., Al-Jaralla, R., & Al-Mahamel, S. (2020). Assessment of air pollutants emissions due to traffic in two residential areas in Kuwait. *International Journal of Environmental Science and Technology,**19,* 807–816. 10.1007/s13762-020-02941-4.

[CR34] Jain, A., Sukhdeve, T., Gadia, H., Sahu, S.P., & Verma, S. (2021). Covid19 prediction using time series analysis. In *2021 International conference on artificial intelligence and smart systems (ICAIS)* (pp. 1599–1606). IEEE.

[CR35] Johansen, S. (1995). *Likelihood-based inference in cointegrated vector autoregressive models*. OUP.

[CR36] Johansen S, Juselius K (1990). Maximum likelihood estimation and inference on cointegration—With applications to the demand for money. Oxford Bulletin of Economics and Statistics.

[CR37] Jones SS, Thomas A, Evans RS, Welch SJ, Haug PJ, Snow GL (2008). Forecasting daily patient volumes in the emergency department. Academic Emergency Medicine.

[CR38] Kočenda, E., & Černỳ, A. (2015). *Elements of time series econometrics: An applied approach*. Charles University in Prague, Karolinum Press.

[CR39] Konarasinghe K (2020). Modeling COVID-19 epidemic of USA, UK and Russia. Journal of New Frontiers in Healthcare and Biological Sciences.

[CR40] Kwiatkowski D, Phillips P, Schmidt P, Shin Y (1992). Distribution of the estimators for autoregressive time series with a unit root. Journal of Econometrics.

[CR41] Latief R, Kong Y, Javeed SA, Sattar U (2021). Carbon emissions in the SAARC countries with causal effects of FDI, economic growth and other economic factors: Evidence from dynamic simultaneous equation models. International Journal of Environmental Research and Public Health.

[CR42] Lauc G, Markotić A, Gornik I, Primorac D (2020). Fighting COVID-19 with water. Journal of Global Health.

[CR43] Liu J, Zhou J, Yao J, Zhang X, Li L, Xu X, He X, Wang B, Fu S, Niu T (2020). Impact of meteorological factors on the COVID-19 transmission: A multi-city study in China. Science of the Total Environment.

[CR44] Ma Y, Zhao Y, Liu J, He X, Wang B, Fu S, Yan J, Niu J, Zhou J, Luo B (2020). Effects of temperature variation and humidity on the death of COVID-19 in Wuhan, China. Science of the Total Environment.

[CR45] Mahadeva, L., & Robinson, P. (2004). *Unit root testing to help model building*. Centre for Central Banking Studies, Bank of England.

[CR46] Martelletti L, Martelletti P (2020). Air pollution and the novel COVID-19 disease: A putative disease risk factor. SN Comprehensive Clinical Medicine.

[CR47] Menebo MM (2020). Temperature and precipitation associate with COVID-19 new daily cases: A correlation study between weather and COVID-19 pandemic in Oslo, Norway. Science of the Total Environment.

[CR48] Murugesan B, Karuppannan S, Mengistie AT, Ranganathan M, Gopalakrishnan G (2020). Distribution and trend analysis of COVID-19 in India: Geospatial approach. Journal of Geographical Studies.

[CR49] Mustafa, H. I., & Fareed, N. Y. (2020). COVID-19 cases in Iraq; Forecasting incidents using Box–Jenkins ARIMA model. In *2020 2nd Al-Noor international conference for science and technology (NICST)* (pp. 22–26). IEEE.

[CR50] Nguyen HM, Turk PJ, McWilliams AD (2021). Forecasting COVID-19 Hospital Census: A multivariate time-series model based on local infection incidence. JMIR Public Health and Surveillance.

[CR51] Ogen Y (2020). Assessing nitrogen dioxide (NO$$_2$$) levels as a contributing factor to coronavirus (COVID-19) fatality. Science of the Total Environment.

[CR52] Pani SK, Lin NH, RavindraBabu S (2020). Association of COVID-19 pandemic with meteorological parameters over Singapore. Science of the Total Environment.

[CR53] Pata UK (2020). How is COVID-19 affecting environmental pollution in us cities? Evidence from asymmetric Fourier causality test. Air Quality, Atmosphere & Health.

[CR54] Pesaran, M. H., & Shin, Y. (1995). An autoregressive distributed lag modelling approach to cointegration analysis.

[CR55] Phillips PC, Perron P (1988). Testing for a unit root in time series regression. Biometrika.

[CR56] Pirouz, B., Golmohammadi, A., Saeidpour Masouleh, H., Violini, G., & Pirouz, B. (2020). Relationship between average daily temperature and average cumulative daily rate of confirmed cases of COVID-19. medRxiv. 10.1101/2020.04.10.20059337.

[CR57] Roy S, Bhunia GS, Shit PK (2021). Spatial prediction of COVID-19 epidemic using ARIMA techniques in India. Modeling Earth Systems and Environment.

[CR58] Sahai AK, Rath N, Sood V, Singh MP (2020). ARIMA modelling & forecasting of COVID-19 in top five affected countries. Diabetes & Metabolic Syndrome: Clinical Research & Reviews.

[CR59] Sharma S, Zhang M, Gao J, Zhang H, Kota SH (2020). Effect of restricted emissions during COVID-19 on air quality in India. Science of the Total Environment.

[CR60] Shehzad K, Sarfraz M, Shah SGM (2020). The impact of COVID-19 as a necessary evil on air pollution in India during the lockdown. Environmental Pollution.

[CR61] Shi P, Dong Y, Yan H, Zhao C, Li X, Liu W, He M, Tang S, Xi S (2020). Impact of temperature on the dynamics of the COVID-19 outbreak in China. Science of the Total Environment.

[CR62] Sobral MFF, Duarte GB, da Penha Sobral AIG, Marinho MLM, de Souza Melo A (2020). Association between climate variables and global transmission of SARS-CoV-2. Science of The Total Environment.

[CR63] Sulasikin, A., Nugraha, Y., Kanggrawan, J., & Suherman, A. L. (2020). Forecasting for a data-driven policy using time series methods in handling COVID-19 pandemic in Jakarta. In *The 6th IEEE international smart cities conference (ISC2 2020)*. 10.1109/ISC251055.2020.9239066.

[CR64] Tosepu R, Gunawan J, Effendy DS, Lestari H, Bahar H, Asfian P (2020). Correlation between weather and COVID-19 pandemic in Jakarta, Indonesia. Science of The Total Environment.

[CR65] Tyagi, R., Bramhankar, M., Pandey, M., & Kishore, M. (2020). COVID 19: Real-time forecasts of confirmed cases, active cases, and health infrastructure requirements for India and its states using the ARIMA model. *medRxiv*. 10.1101/2020.05.17.20104588.

[CR66] Wang, W. (2006). *Stochasticity, nonlinearity and forecasting of streamflow processes*. Ios Press.

[CR67] Wu F, Zhao S, Yu B, Chen YM, Wang W, Song ZG, Hu Y, Tao ZW, Tian JH, Pei YY (2020). A new coronavirus associated with human respiratory disease in China. Nature.

[CR68] Xie J, Zhu Y (2020). Association between ambient temperature and COVID-19 infection in 122 cities from China. Science of the Total Environment.

[CR69] Yonar H, Yonar A, Tekindal MA, Tekindal M (2020). Modeling and forecasting for the number of cases of the COVID-19 pandemic with the curve estimation models, the Box–Jenkins and exponential smoothing methods. EJMO.

[CR70] Zhu Y, Xie J, Huang F, Cao L (2020). Association between short-term exposure to air pollution and COVID-19 infection: Evidence from China. Science of the Total Environment.

