# Correction to: Exploring the impact of air pollution on COVID-19 admitted cases: Evidence from vector error correction model (VECM) approach in explaining the relationship between air pollutants towards COVID-19 cases in Kuwait

**DOI:** 10.1007/s42081-022-00174-y

**Published:** 2022-08-18

**Authors:** Ahmad R. Alsaber, Parul Setiya, Ahmad T. Al-Sultan, Jiazhu Pan

**Affiliations:** 1 Department of Management, American University of Kuwait, Salmiya, Kuwait; 2 Department of Agrometeorology, College of Agriculture, G.B.Pant University of Agriculture and Technology, Pantnagar, Uttarakhand India; 3 Department of Community Medicine and Behavioural Sciences, College of Medicine, Kuwait University, Kuwait City, Kuwait; 4 Department of Mathematics and Statistics, University of Strathclyde, Glasgow, G1 1XH UK

## Correction to: Japanese Journal of Statistics and Data Science (2022) 5:379–406 10.1007/s42081-022-00165-z

In the original publication, the affiliation citation appears incorrectly. The corrected affiliation of the authors should read as:

Ahmad R. Alsaber^1^, Parul Setiya^2^, Ahmad T. Al-Sultan^3^, Jiazhu Pan.^4^

Ahmad R. Alsaber (first author)

Department of Management, American University of Kuwait, Salmiya, Kuwait.

Parul Setiya (second author)

Department of Agrometeorology, College of Agriculture G.B.Pant University of Agriculture & Technology, Pantnagar, Uttarakhand, India.

Ahmad T. Al-Sultan (Third author)

Department of Community Medicine and Behavioural Sciences, College of Medicine, Kuwait University.

Jiazhu Pan (Fourth Author)

Department of Mathematics and Statistics, University of Strathclyde Glasgow, G1 1XH, UK.

The original article has been corrected.

